# All-Day Thermogalvanic Cells for Environmental Thermal Energy Harvesting

**DOI:** 10.34133/2019/2460953

**Published:** 2019-10-31

**Authors:** Boyang Yu, Jiangjiang Duan, Jia Li, Wenke Xie, Hongrun Jin, Rong Liu, Hui Wang, Liang Huang, Bin Hu, Jun Zhou

**Affiliations:** Wuhan National Laboratory for Optoelectronics, Huazhong University of Science and Technology, Wuhan 430074, China

## Abstract

Direct conversion of the tremendous and ubiquitous low-grade thermal energy into electricity by thermogalvanic cells is a promising strategy for energy harvesting. The environment is one of the richest and renewable low-grade thermal source. However, critical challenges remain for all-day electricity generation from environmental thermal energy due to the low frequency and small amplitude of temperature fluctuations in the environment. In this work, we report a tandem device consisting of a polypyrrole (PPy) broadband absorber/radiator, thermogalvanic cell, and thermal storage material (Cu foam/PEG1000) that integrates multiple functions of heating, cooling, and recycling of thermal energy. The thermogalvanic cell enables continuous utilization of environmental thermal energy at both daytime and nighttime, yielding maximum outputs as high as 0.6 W m^−2^ and 53 mW m^−2^, respectively. As demonstrated outdoors by a large-scale prototype module, this design offers a feasible and promising approach to all-day electricity generation from environmental thermal energy.

## 1. Introduction

Low-grade thermal energy (<100°C) is an energy source with tremendous potential that exists in the environment, industrial processes, and the human body [1–3]. Unfortunately, most of this energy is wasted due to wide distribution and limited recovery technologies [4, 5] as well as the consumption of extra energy for dissipation, which is harmful to global energy conservation and cooling. Direct conversion of low-grade thermal energy into electricity by thermoelectric technologies, without any energy consumption or carbon emission, is a promising strategy for the imminent energy and environmental crises [6]. Conventional solid-state thermoelectric devices have high efficiency at high temperatures, but high costs and material limitations impede their practical application for low-grade thermal energy [7–9].

Thermogalvanic cells (TGCs) that consist of redox couples, electrolytes, and electrodes can generate sustainable electricity due to a temperature-dependent redox potential [10–12]. The features of TGCs, including a high Seebeck coefficient (*S*_*e*_) (~1 mV K^−1^), low cost, flexibility, scalable route, and matched operation temperature, make these cells an ideal alternative to solid-state thermoelectric devices for large-scale low-grade thermal energy harvesting [13]. For TGC systems, the open-circuit voltage (*V*_oc_) is described as follows [3]:

(1) $$\begin{array}{c} V_{\text{oc}}=S_{e}\times \Delta T, \end{array}$$

where Δ*T* is the temperature differential. Obviously, a real-time spatial temperature differential is absolutely necessary for electricity generation. In the practical scenarios, the operated Δ*T* is mostly yielded between heat sources and an ambient environment [3, 14, 15]. However, it is generally ignored that the environment itself is one of the most abundant and renewable low-grade thermal energy sources. Environmental thermal energy is present in the form of fluctuations of environmental temperature over time (e.g., diurnal fluctuation) [16], mainly contributed by earth absorbing solar irradiation at daytime and passively radiating heat to the outer space at nighttime and affected by ever-changing weather conditions, different seasons, and locations. Unfortunately, due to the single temporal temperature differential, all-day electricity generation from environmental thermal energy remains a critical challenge. To harvest temperature fluctuations for electricity generation, some novel and emerging technologies have been reported and developed recently, such as pyroelectric energy harvesters [17–19], thermally regenerative electrochemical cycles [2, 6, 20], and thermal resonators [16, 21]. However, pyroelectric energy harvesters strongly rely on high-frequency temperature fluctuations [18], mismatching the wide diurnal fluctuation of environmental temperature. Although thermally regenerative electrochemical cycles exhibit high efficiency with a small-scale device, the high cost, electrode reversibility, and cell durability still limit their application at large scale [5]. Thermal resonators provide an approach to the conversion of temporal temperature differential to spatial temperature differential by using phase change materials (PCMs) and have the capability of being optimized at different target frequencies of temperature fluctuations [16], but the small amplitude (generally approximate to the temperature difference between day and night) of temperature fluctuations becomes a critical limitation when they are applied in practical environmental thermal energy harvesting. Not only the low frequency but also the small amplitude of temperature fluctuations impedes effective utilization of environmental thermal energy by current single technology. It is worth noting that solar irradiation is a significant contributor of environmental temperature fluctuations and solar-thermal conversion technologies have been extensively investigated for solar steam generation [22–27], electricity generation [28–32], and solar hot-water systems [33]. In addition, passive radiative cooling (PRC), a phenomenon in which a surface spontaneously cools by radiating heat to the cold outer space through the longwave infrared (LWIR) transmission window (8-13 *μ*m) of the atmosphere, has been demonstrated to supply considerable cooling power density without sunlight [34–38]. Hence, the development of hybrid systems might introduce a novel avenue for the use of environmental thermal energy in all-day electricity generation, which is of great importance to relieve energy issues.

**Figure 1:**
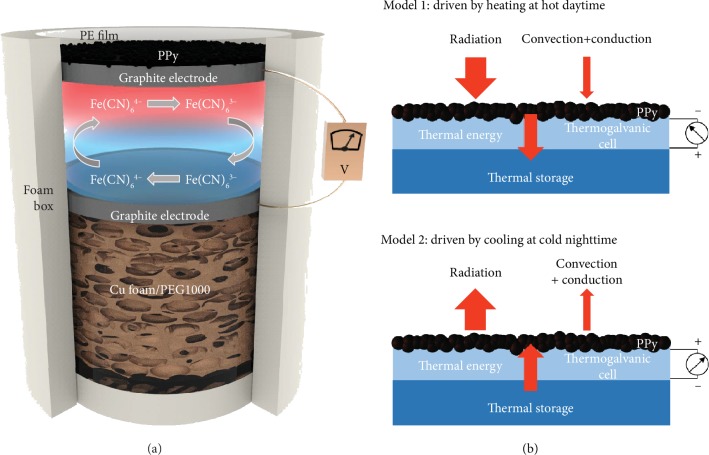
Schematic of the device for all-day low-grade environmental thermal energy harvesting. (a) Tandem structure of the thermogalvanic cell. (b) Schematic mechanisms of two working models of the thermogalvanic cell and corresponding energy flux. Model 1 (upper) and model 2 (lower) work at daytime and nighttime, respectively.

In this work, we report a tandem device based on a polypyrrole (PPy) broadband absorber/radiator layer, thermogalvanic cell, and thermal storage material that maximizes the temperature differential (Δ*T*) across the device during the traditional small amplitude of environmental temperature fluctuations and achieves all-day electricity generation. The structure of the thermogalvanic cell is illustrated in Figure 1(a) and [Supplementary-material supplementary-material-1]. The top layer is a hierarchically structural PPy layer that serves as a heat exchanger with an ambient environment including heating and cooling. The thermogalvanic cell in the middle consists of two graphite sheet electrodes and 0.4 M potassium ferricyanide/ferrocyanide (K_3_Fe(CN)_6_/K_4_Fe(CN)_6_) aqueous electrolyte with a relatively high Seebeck coefficient (*S*_*e*_) of ~-1.4 mV K^−1^ [1, 39]. A PCM (labelled as Cu foam/PEG1000 in Figure 1(a) and [Supplementary-material supplementary-material-1]) at the bottom stores thermal energy and maintains a hysteretic temperature (near the phase transition temperature *T*^∗^) on the bottom electrode.

The mechanisms of the two working models of the device and the corresponding energy flux are schematically depicted in Figure 1(b). Model 1 (upper) is driven by heating at daytime with a relatively hot environmental temperature and sometimes natural sunlight. The top electrode achieves a high temperature via the PPy layer absorbing radiation from an ambient environment and natural sunlight, whereas the bottom electrode maintains a low temperature by storing latent heat in the PCM, yielding a large temperature differential (Δ*T*) across the TGC. Complementary model 2 (lower) is driven by cooling at nighttime with a relatively cold environmental temperature. The top electrode cools quickly due to the strong radiative cooling ability of the PPy layer, and the bottom electrode also maintains the temperature near the phase change temperature (*T*^∗^) of the PCM. As a result, a considerable inverse Δ*T* is built in the TGC. Consequently, the thermogalvanic cell with a large Δ*T* yields an impressive maximum output of 0.6 W m^−2^ at sunny daytime, and an extra output of 53 mW m^−2^ is still achieved at nighttime. In addition, the device also exhibits a continuous output during ambient environmental temperature fluctuation without any illumination, which testify its feasibility at sunless day. Furthermore, a proof-of-concept large-scale prototype is successfully fabricated to demonstrate the ability to harvest and recycle environmental thermal energy for all-day electricity generation outdoors as well as the feasibility of scale up.

## 2. Results and Discussion

### 2.1. Characterizations of the Polypyrrole- (PPy-) Modified Graphite Sheet

**Figure 2:**
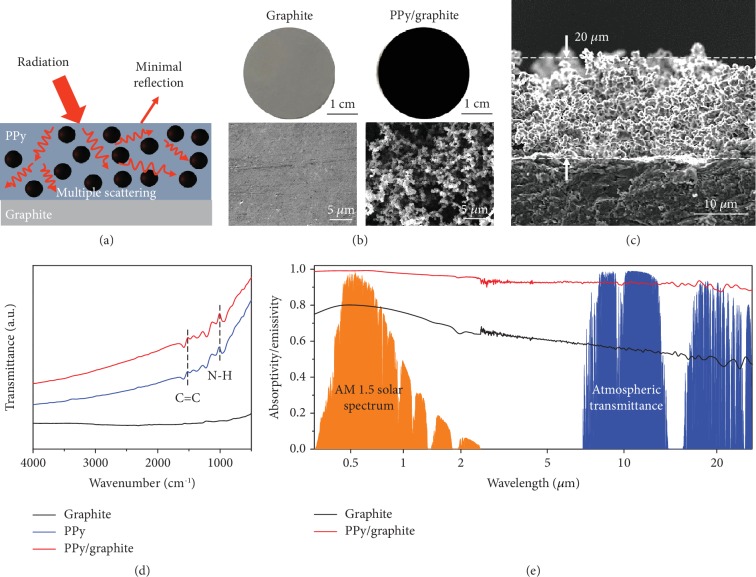
Characterizations of the polypyrrole- (PPy-) modified graphite sheet. (a) Schematic of multiple scattering of radiation in the nano-/microstructure of PPy. (b) Photographs and SEM images of pristine graphite sheet (graphite) and PPy-modified graphite sheet (PPy/graphite). (c) Cross-sectional SEM of PPy/graphite. The average thickness of PPy on the graphite sheet is approximately 20 *μ*m. (d) FTIR spectrum of PPy/graphite, pristine PPy, and graphite. (e) Measured absorptivity/emissivity spectrum of PPy/graphite and graphite in both the solar (0.3 to 2.5 *μ*m) and infrared (2.5 to 25 *μ*m) regions, with the standard AM1.5 solar spectrum (shaded orange) and atmospheric transmittance *t*(*λ*) (shaded blue) plotted for reference [42].

We used the in situ chemical oxidation method to polymerize PPy on the top graphite electrode (see Supplementary Materials for details). The graphite sheets were selected as the electrodes for the TGC due to its low cost and relatively high current density [3].  Figure 2(b) compares the optical photograph and corresponding surface scanning electron microscopy (SEM) image of a PPy-modified graphite sheet (labelled as PPy/graphite) with those from a pristine graphite sheet (labelled as graphite). The PPy/graphite is notably dark in contrast to the pristine light-grey graphite, and PPy displays a typically cauliflower-like hierarchical structure ranging from nanosize to microsize. The cross-sectional SEM image (Figure 2(c)) shows the PPy layer with an average thickness of 20 *μ*m on the graphite sheet. The dependence of the thickness of PPy on polymerization times was also characterized by SEM ([Supplementary-material supplementary-material-1]). The chemical composition of the PPy/graphite was analysed by Fourier transform infrared (FTIR) spectroscopy (Figure 2(d)). The spectrum of PPy/graphite shows identical absorption peaks at 1517 cm^−1^ and 1014 cm^−1^, corresponding to the in-ring stretching of C=C bonds in the pyrrole rings and the in-plane deformation of N–H bonds, respectively [40]. No absorption peak is present for graphite (Figure 2(d)). Furthermore, we also investigated the stability of PPy/graphite via FTIR spectroscopy and thermogravimetric analysis (TGA), as shown in [Supplementary-material supplementary-material-1]. All of the characteristic peaks of PPy are consistent with the pristine sample after exposure to the environment for one month, indicating excellent stability for outdoor operation.

As schematically depicted in Figure 2(a), the mechanism benefits from the varied sizes of the PPy clusters and matched bonding frequency and multiple scattering and absorption of radiation exist in the hierarchical PPy layer that significantly suppresses reflection. Therefore, PPy/graphite exhibits ultrahigh broadband absorptivity/emissivity, showing distinct advantages over pristine graphite. The spectroscopic performance in both the solar (0.3 to 2.5 *μ*m) and infrared (2.5 to 25 *μ*m) regions was characterized by ultraviolet-visible-near-infrared (UV-Vis-NIR) spectrophotometry and FTIR spectrometry, respectively (Figure 2(e)). The absorptivity of PPy/graphite is greater than 0.98, as weighted by the standard air mass 1.5 global (AM 1.5 G) solar spectrum. The average emissivity of approximately 0.93 is measured over the atmospheric LWIR transmission window (8-13 *μ*m). Both of these values lay the foundation for efficient heating at daytime and cooling at nighttime. Furthermore, we compared the absorptivity/emissivity values of different PPy thickness samples ([Supplementary-material supplementary-material-1]), which were nearly equal within the range of errors. Hence, PPy/graphite with a PPy thickness of 20 *μ*m was used in the following experiments, considering the relatively low thermal resistance.

### 2.2. Performances of Heating and Cooling

**Figure 3:**
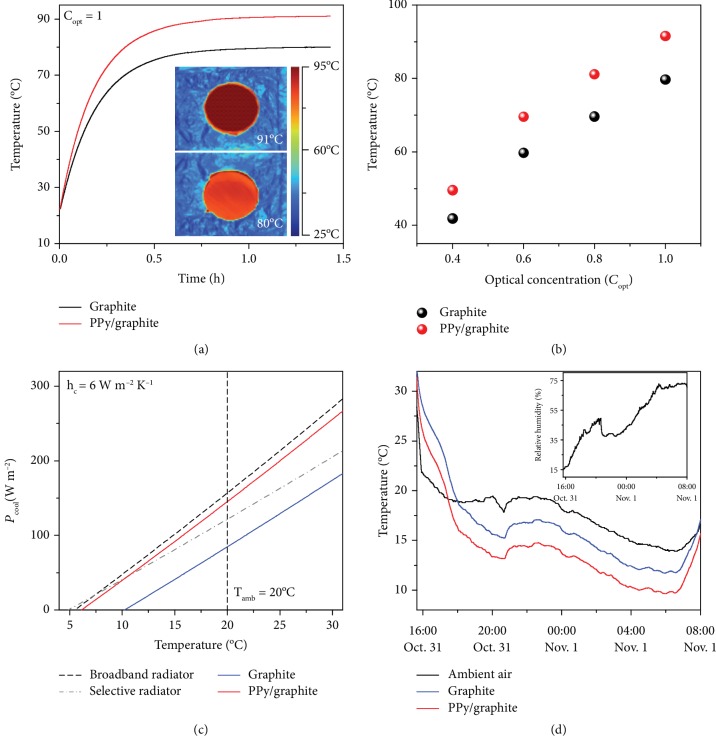
Performances of heating and cooling. (a) Increasing temperature curves of PPy/graphite and graphite under illumination with *C*_opt_ = 1. The insets show the corresponding infrared (IR) thermal images at a steady state. (b) Steady-state temperature of PPy/graphite and graphite under different optical concentrations. (c) Net cooling power *P*_cool_ versus radiator temperature for PPy/graphite, graphite, a broadband radiator, and a selective radiator simulated by considering a nonradiative heat coefficient (*h*_*c*_) of 6 W m^−2^ K^−1^ [43]. (d) A 16-hour continuous temperature measurement of PPy/graphite, graphite, and ambient air from 16:00 (October 31, 2018) to 8:00 (November 1, 2018) at the Wuhan National Laboratory for Optoelectronics (WNLO), China (30°30′49′′ N, 114°25′13′′ E, 35 m altitude). The inset shows the ambient relative humidity fluctuation.

To test the performance of heating assisted with natural sunlight, the PPy/graphite and graphite were illuminated with different energy densities generated by a solar simulator. As shown in Figure 3(a), the temperature of the samples increases with the increase in illumination time. Due to the excellent absorptivity, as noted above, PPy/graphite exhibits a more rapid rate of temperature increase and reaches a steady-state temperature of 91°C under one solar radiation density, much higher than that of graphite at 80°C, in agreement with infrared (IR) thermal images of the steady state (inset of Figure 3(a)). In addition, the steady-state temperature of PPy/graphite at different illumination densities is significantly higher than that of graphite (Figure 3(b) and [Supplementary-material supplementary-material-1]). These results verify the critical role of the hierarchical PPy layer in enhancing heating ability.

The radiative cooling performance of PPy/graphite and graphite were also investigated by theoretical simulations and outdoor experiments. Considering all of the heat exchange processes, the net cooling power (*P*_cool_) of a radiator can be defined as follows [34]:

(2) $$\begin{array}{c} P_{\text{cool}}\left(T\right)=P_{\text{rad}}\left(T\right)-P_{\text{atm}}\left(T_{\text{amb}}\right)-P_{\text{sun}}-P_{\text{cond}+\text{conv}}, \end{array}$$

where

(3) $$\begin{array}{c} P_{\text{rad}}\left(T\right)=2\pi \int_{0}^{\pi /2}\sin\theta \cos\theta d\theta \int_{0}^{\infty }I_{BB}\left(T,\lambda \right)\epsilon \left(\lambda ,\theta \right)d\lambda \end{array}$$

is the radiation emitted by the radiator,

(4) $$\begin{array}{c} P_{\text{atm}}\left(T_{\text{amb}}\right)=2\pi \int_{0}^{\pi /2}\sin\theta \cos\theta d\theta \int_{0}^{\infty }I_{BB}\left(T_{\text{amb}},\lambda \right)\epsilon \left(\lambda ,\theta \right)\epsilon_{\text{atm}}\left(\lambda ,\theta \right)d\lambda \end{array}$$

is the incident atmospheric radiation absorbed by the radiator,

(5) $$\begin{array}{c} P_{\text{cond}+\text{conv}}\left(T,T_{\text{amb}}\right)=h_{c}\left(T_{\text{amb}}-T\right) \end{array}$$

is the thermal losses due to convection and conduction and *P*_sun_ is the incident solar power absorbed by the radiator.

In this work, *I*_*BB*_(*T*, *λ*) = (2*hc*^2^/*λ*^5^)(1/(*e*^*hc*/(*λk*_*B*_*T*)^ − 1)) is the spectral radiance of a blackbody defined by Planck's law at temperature *T*, where *h* is Planck's constant, *k*_*B*_ is the Boltzmann constant, *c* is the speed of light in a vacuum, *λ* is the wavelength, and *ϵ*(*λ*, *θ*) is the emissivity of the radiator according to Kirchhoff's law. The angle-dependent emissivity of the atmosphere is given by [41] *ϵ*_atm_(*λ*, *θ*) = 1 − *t*(*λ*)^1/cos*θ*^, where *t*(*λ*) is the atmospheric transmittance in the zenith direction [42], *T* and *T*_amb_ are the temperatures of the radiator and ambient air, respectively, and *h*_*c*_ = *h*_cond_ + *h*_conv_ is a combined nonradiative heat coefficient stemming from the conductive and convective heat exchange of the radiator with the ambient air.

Considering the practical operation of PPy/graphite and graphite at night, we assumed the terms *P*_sun_ = 0, *T*_amb_ = 20°C, and *h*_*c*_ = 6 W m^−2^ K^−1^ [43]. The simulated *P*_cool_ of PPy/graphite, graphite, an ideal broadband radiator (i.e., blackbody), and an ideal selective radiator (which has a unity emissivity only over the atmospheric LWIR transmission window of 8-13 *μ*m) are shown in Figure 3(c). The transverse intercept (*P*_cool_ = 0) represents the lowest temperature that the radiator can reach. The ideal selective radiator can reach a lower temperature, whereas it has an inferior *P*_cool_ when the temperature is not much lower than *T*_amb_ [44]. In contrast, the ideal broadband radiator has a superior *P*_cool_ over a wide temperature range, especially at high temperature. In this work, the device is heated by thermal storage materials at night (Figure 1(b)), the temperature of which is higher than *T*_amb_ at all times. Therefore, the ideal broadband radiator is a better choice. Because the high emissivity in the entire infrared band is close to that of the ideal broadband radiator, the PPy/graphite exhibits much higher *P*_cool_ than graphite. Furthermore, we demonstrated the real-time, continuous outdoor radiative cooling performances of the samples after solar heating (Figure 3(d)). In addition, the fluctuation of relative humidity in the ambient air was also measured (inset of Figure 3(d)). PPy/graphite yields an average of ~2.5°C and ~5°C lower than graphite and ambient air, respectively. The remarkable heating and cooling performance of PPy/graphite is expected to generate as much larger Δ*T* for TGC operation than graphite in the day and at night, respectively.

### 2.3. Performance of Electricity Generation

Although a highly efficient heat exchanger assisted with solar heating and radiative cooling is used, the Δ*T* across the TGC is still limited by the synchronous temperature fluctuations of both the top and bottom electrodes. To achieve a larger Δ*T* and recycle the residual thermal energy simultaneously, we connected the Cu foam/PEG1000 to the bottom electrode of the TGC. The Cu foam serves as a highly thermally conducting and porous matrix [16], and the PEG1000 bolsters the thermal capacitance through the latent heat of its phase change. PEG1000 is chosen as the PCM due to its suitable phase transition temperature (*T*^∗^, 38°C), which is approximately the average temperature of the device during all-day operation ([Supplementary-material supplementary-material-1]). The Cu foam/PEG1000 with a high thermal effusivity (*e*) (see Supplementary [Supplementary-material supplementary-material-1] and [Supplementary-material supplementary-material-1]) not only stores residual thermal energy via phase transition but also maintains the temperature of the bottom electrode near *T*^∗^. The stored thermal energy is recycled as a heat source during night-time operation. Via the synergistic effect of PPy and Cu foam/PEG1000, a considerable Δ*T* (which means high output) can be yielded easily both in the daytime and at night without any complex optical or thermal concentration systems.

**Figure 4:**
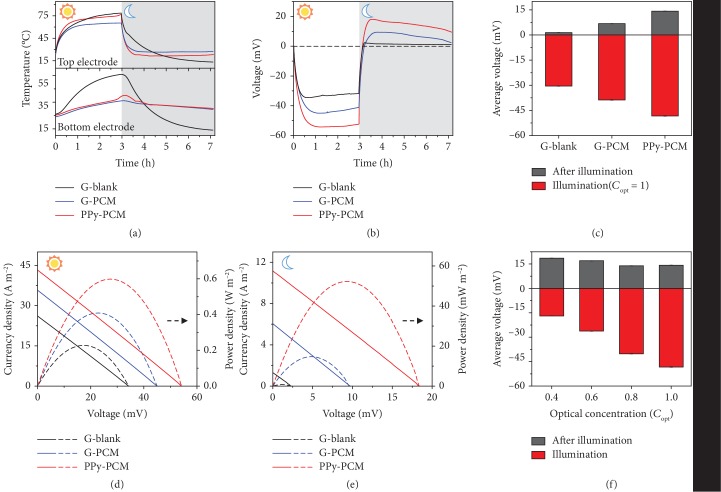
Performance of electricity generation. (a) Temperature curves of the top electrodes (upper) and the bottom electrodes (lower). (b, c) Open-circuit voltage (*V*_oc_) curves (b) and average *V*_oc_ (c) of PPy-PCM, G-PCM, and G-blank versus time under illumination with *C*_opt_ = 1 (white area) and after illumination (gray area). (d, e) Maximum current-voltage and power-voltage curves of PPy-PCM, G-PCM, and G-blank under illumination with *C*_opt_ = 1 (d) and after illumination (e), respectively. (f) Average *V*_oc_ of PPy-PCM under different optical concentrations.

To verify the rationality of our design, we compared the output performances of three different devices, namely, PPy-PCM (using both PPy/graphite and PCM), G-PCM (using graphite and PCM), and G-blank (using only graphite). During operation, all of the devices were illuminated to simulate the environment of sunny day, and the corresponding open-circuit voltage (*V*_oc_) and the temperatures of the top electrodes (*T*_top_) and the bottom electrodes (*T*_bottom_) were recorded. As shown in Figure 4(a), the *T*_top_ of PPy-PCM increases more rapidly and reaches a higher steady-state temperature than that of G-PCM due to better heating performance. The *T*_top_ of PPy-PCM is a little lower than that of G-blank owing to its much lower *T*_bottom_. The *T*_bottom_ values of PPy-PCM and G-PCM both increase slowly near *T*^∗^ (38°C) compared with that of G-blank, which is ascribed to the phase transition of the Cu foam/PEG1000. As a result, the largest Δ*T* is measured in PPy-PCM under illumination. Corresponding to the regularity of temperature, PPy-PCM yields a maximum negative *V*_oc_ of -54.2 mV, much larger than those of G-PCM (-44.9 mV) and G-blank (-34.1 mV), as clearly shown in Figure 4(b). When the phase change of PCM was complete (approximately three hours of illumination), the illumination was turned off and the devices were exposed to a mixture of ice water (~273 K) without direct contact (exchanging heat only by radiation) to simulate radiative cooling at a night-time environment. As shown in the grey area of Figure 4(a), it is worth noting that the superior radiative cooling ability of PPy-PCM produces a much lower *T*_top_ compared with that of G-PCM. The *T*_top_ of PPy-PCM is higher than that of G-blank owing to its much higher *T*_bottom_. The *T*_bottom_ values of PPy-PCM and G-PCM have a long-term hysteresis effect and are higher than *T*_top_ due to the release of latent heat by the Cu foam/PEG1000. Consequently, a maximum positive *V*_oc_ of 18.2 mV is also achieved by PPy-PCM without illumination, which is almost twice that of G-PCM at 9.2 mV (Figure 4(b)). Without PCM, the Δ*T* of G-blank driven by the weak radiative cooling is so small that it only generates a positive *V*_oc_ of less than 2.2 mV. Furthermore, we counted the average *V*_oc_ of these three devices under illumination (*C*_opt_ = 1) and after illumination (Figure 4(c)). Obviously, PPy-PCM generates the highest voltage output regardless of illumination and darkness.

The current-voltage curves measured at the maximum *V*_oc_ and the corresponding output power density are shown in Figure 4(d) (under illumination) and Figure 4(e) (after illumination). The short-circuit current (*I*_sc_) and maximum power density (*P*_max_) of PPy-PCM reach 43.5 A m^−2^ and 0.6 W m^−2^ under illumination, and the considerable *I*_sc_ of 11.5 A m^−2^ and *P*_max_ of 53 mW m^−2^ are still yielded even after illumination. In comparison, the corresponding values of G-PCM are 35.7 A m^−2^ and 0.4 W m^−2^ and 6 A m^−2^ and 14 mW m^−2^, respectively, and the G-blank reaches only 25.9 A m^−2^ and 0.22 W m^−2^ and 1.3 A m^−2^ and 0.8 mW m^−2^, respectively. It is unquestionable that PPy-PCM is the best choice for solar thermal energy harvesting. To estimate the feasibility of PPy-PCM in various weather conditions, we further tested its performance under varying optical concentration illumination and after illumination ([Supplementary-material supplementary-material-1]). The calculated average *V*_oc_ values in different conditions are shown in Figure 4(f). With the increase in optical concentration, the average *V*_oc_ under illumination increases accordingly, whereas the average *V*_oc_ after illumination changes with small fluctuation due to the same storage thermal energy by PCM. Furthermore, we calculated the total efficiency (*η*_total_) for PPy-PCM, representing 50% and 200% enhancements of those of G-PCM and G-blank, respectively (see Supplementary [Supplementary-material supplementary-material-1] and [Supplementary-material supplementary-material-1]).

Considering the sunless day during the practical scenarios, the PPy-PCM device was exposed to a hot and cold environment without illumination successively to test its performance of all-day electricity generation. As shown in [Supplementary-material supplementary-material-1], the device generates *V*_oc_ continuously from a hot ambient temperature (45°C) to a cold ambient temperature (15°C). The maximum negative and positive *V*_oc_ are -12 mV and 10 mV, respectively. And the corresponding *I*_sc_ and *P*_max_ are 7.6 A m^−2^ and 24 mW m^−2^and 6.4 A m^−2^ and 17 mW m^−2^ ([Supplementary-material supplementary-material-1]).

### 2.4. Outdoor Demonstration of a Large-Scale Prototype Module

**Figure 5:**
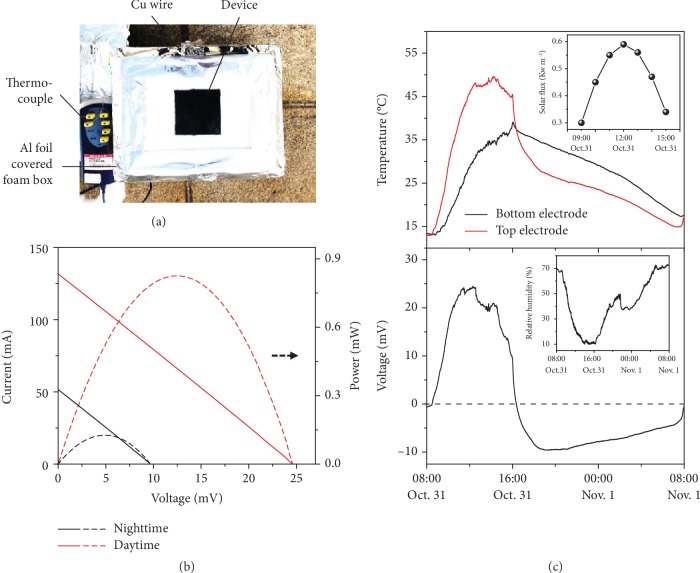
Outdoor demonstration of a large-scale prototype module. (a) Photograph of the thermogalvanic cell prototype with an active area of 100 cm^2^. The device is fixed by an aluminum foil-covered foam box to decrease thermal losses and avoid sunlight absorption around the device. (b) Maximum current-voltage and power-voltage curves of the device at daytime and nighttime, respectively. (c) Temperature (upper) of the top electrode and bottom electrode and open-circuit voltage (lower) versus time. The upper and lower insets show the measured natural solar intensity and ambient relative humidity, respectively. The device operated continuously from 8:00 (October 31, 2018) to 8:00 (November 1, 2018) at the Wuhan National Laboratory for Optoelectronics (WNLO), China (30°30′49′′ N, 114°25′13′′ E, 35 m altitude).

To demonstrate the practical applications of this design for all-day harvesting of environmental thermal energy, a proof-of-concept tandem thermogalvanic cell prototype was fabricated for outdoor testing (Figure 5(a)). The device is based on a large-scale PPy/graphite with an active area of 10 cm × 10 cm ([Supplementary-material supplementary-material-1]). The used volume of the Cu foam/PEG1000 is simulated and depends on the absorption of all of the residual thermal energy during the daytime (see Supplementary [Supplementary-material supplementary-material-1] and [Supplementary-material supplementary-material-1]). We measured the 24-hour continuous open-circuit voltage (*V*_oc_) of the device and the temperatures of the top electrode (*T*_top_) and the bottom electrode (*T*_bottom_). Additionally, the solar flux of natural sunlight in the day and the relative humidity in the ambient air were also recorded. As shown in Figure 5(c), the *T*_top_ and the *T*_bottom_ increase synchronously with the enhancement of solar intensity and ambient temperature in the morning, reach a maximum at noon, and decrease after sunset. However, the impressive heating and cooling performance of the PPy layer mean that the *T*_top_ is much higher at daytime with natural sunlight and is much lower at nighttime, respectively. The *T*_bottom_ clearly exhibits a long-term hysteresis effect near *T*^∗^ (38°C) due to the phase transition of the Cu foam/PEG1000. Consequently, a considerable Δ*T* across the device lasts from day to night to generate a sustainable *V*_oc_ (bottom graph in Figure 5(c)). The *V*_oc_ reaches approximately 24.7 mV at daytime (average solar flux of ~0.5 kW m^−2^, upper inset of Figure 5(c)) and 9.8 mV at nighttime, and the corresponding *I*_*sc*_ values are approximately 134 mA and 52 mA, resulting in maximum output power values of 0.83 mW and 0.13 mW, respectively (Figure 5(b)).

## 3. Conclusions

In summary, a tandem device consisting of a absorber/radiator layer (PPy), a thermogalvanic cell, and a thermal storage material (Cu foam/PEG1000) was designed to harness and recycle environmental thermal energy for all-day electricity generation. The PPy layer with ultrahigh broadband absorptivity/emissivity exhibits impressive performance in heating at daytime and cooling at nighttime. The reversible phase transition processes of the Cu foam/PEG1000 enable the thermogalvanic cell to recycle residual thermal energy and generate electricity day and night, regardless of the single temporal temperature differential existing in an environment. By the synergistic enhancement of PPy/graphite and Cu foam/PEG1000, the thermogalvanic cell yielded a maximum electrical output power of 0.6 W m^−2^ at daytime with simulated sunlight and 53 mW m^−2^ at nighttime. Even at the sunless environment, the thermogalvanic cells also exhibit the ability of continuous electricity generation, which opens a promising path to enhance environmental thermal energy harvesting. In addition, the performance of the device can be further improved using a TGC with high Seebeck coefficient and optimized electrodes [1, 3, 39, 45].

## Supplementary Materials

Supplementary Materials Supplementary Experimental Procedures: (1) materials, (2) polypyrrole-modified graphite sheet (PPy/graphite) preparation, (3) thermal storage material (Cu foam/PEG1000) preparation, (4) electrolyte solution preparation, (5) fabrication of thermogalvanic cells and large-scale prototype module, (6) material characterization, and (7) device characterization. Supplementary Notes: (1) thermal storage material (Cu foam/PEG1000) simulations and (2) energy efficiency analysis. Figure S1: photographs of the thermogalvanic cell. Figure S2: characterization of the thickness of PPy. Figure S3: chemical and thermal stability of PPy. Figure S4: absorptivity/emissivity spectrum of PPy/graphite with different PPy thicknesses. Figure S5: performance of heating for PPy/graphite and graphite. Figure S6: characterization and simulation of Cu foam/PEG1000. Figure S7: performance of the device labelled as PPy-PCM. Figure S8: efficiency analysis of the thermogalvanic generator. Figure S9: performance of PPy-PCM exposed to hot and cold environment without illumination successively. Figure S10: preparation for a large-scale prototype module.
