# Supplementary material for: All-Day Thermogalvanic Cells for Environmental Thermal Energy Harvesting
Source: Research (Wash D C). 2019 Oct 31;2019:2460953. doi: 10.34133/2019/2460953 (PMC6944521; doi:10.34133/2019/2460953)
Supplement: Supplementary Materials — Supplementary Experimental Procedures: (1) materials, (2) polypyrrole-modified graphite sheet (PPy/graphite) preparation, (3) thermal storage material (Cu foam/PEG1000) preparation, (4) electrolyte solution preparation, (5) fabrication of thermogalvanic cells and large-scale prototype module, (6) material characterization, and (7) device characterization. Supplementary Notes: (1) thermal storage material (Cu foam/PEG1000) simulations and (2) energy efficiency analysis. Figure S1: photographs of the thermogalvanic cell. Figure S2: characterization of the thickness of PPy. Figure S3: chemical and thermal stability of PPy. Figure S4: absorptivity/emissivity spectrum of PPy/graphite with different PPy thicknesses. Figure S5: performance of heating for PPy/graphite and graphite. Figure S6: characterization and simulation of Cu foam/PEG1000. Figure S7: performance of the device labelled as PPy-PCM. Figure S8: efficiency analysis of the thermogalvanic generator. Figure S9: performance of PPy-PCM exposed to hot and cold environment without illumination successively. Figure S10: preparation for a large-scale prototype module. [file 2460953.f1.docx]

**Supplementary Materials**

**All-day thermogalvanic cells for environmental thermal energy harvesting**

Boyang Yu^†^, Jiangjiang Duan^†^, Jia Li, Wenke Xie, Hongrun Jin, Rong Liu, Hui Wang, Liang Huang, Bin Hu, and Jun Zhou^*^

Wuhan National Laboratory for Optoelectronics, Huazhong University of Science and Technology, Wuhan 430074, China

^*^Correspondence should be addressed to Jun Zhou; [jun.zhou@mail.hust.edu.cn](mailto:jun.zhou@mail.hust.edu.cn)

^†^These people contributed equally to this work

**Supplementary Experimental Procedures**

**Materials**

Potassium ferricyanide (K_3_Fe(CN)_6_), potassium ferrocyanide (K_4_Fe(CN)_6_), polyethylene glycol 1000 (PEG1000), pyrrole (Py, monomer), ferric chloride (FeCl_3_, oxidant), and benzenesulfonic acid (BSA, surfactant) were purchased from Sigma-Aldrich Trading Co. Ltd. and used without further purification. Graphite sheets with an electrical resistivity of approximately 10 µΩ m were commercial products purchased from Graphite Material Company Ltd. Copper (Cu) foams (porosity 95%, 100 ppi, thickness of 2 mm) were commercial products purchased from Metal Foam Material Company Ltd. Deionized (DI) water obtained from a Milli-Q system (Simplicity, Millipore, France) was used in all experiments.

**Polypyrrole-modified graphite sheet (PPy/graphite) preparation**

The in situ chemical oxidation method was used to polymerize PPy on the top surface of the graphite sheets. First, 0.35 mL Py and 0.79 g BSA were dissolved in 100 mL DI water by sonication for 10 min and labelled as solution A. An amount of 0.81 g FeCl_3_ (1:1 with Py in molar weight) was dissolved in 2 mL DI water and labelled as solution B. Both solution A and solution B were subsequently cooled and mixed together with stirring. Only one surface of the pre-plasma graphite sheet was immersed into the mixed solution for polymerization. The polymerization process was conducted at 5 °C for 6 h. The obtained PPy/graphite was washed several times with DI water to remove the residuum. Finally, the purified PPy/graphite was dried at 40 °C in a vacuum oven for 2 h and used in the following experiments. In addition, samples with different thicknesses were prepared by repeating the above polymerization process 1, 2, 3, and 4 times.

**Thermal storage material (****Cu foam/PEG1000) preparation** [1]

The Cu foam/PEG1000 was assembled by vacuum impregnation of the copper (Cu) foam with molten PEG1000 at 60 °C in a vacuum oven for 2 h. The samples were cooled and dried at ambient conditions. Finally, the obtained Cu foam/PEG1000 was tightly stacked and sealed in a container for subsequent uses.

**Electrolyte solution preparation**

For preparation of the 0.4 M K_3_Fe(CN)_6_/K_4_Fe(CN)_6_ electrolyte, 65.8 g K_3_Fe(CN)_6_ and 84.5 g K_4_Fe(CN)_6_ were dissolved into 500 mL of water with stirring.

**Fabrication of thermogalvanic cells and** **large-scale prototype module**

A typical thermogalvanic cell (TGC) was fabricated with two graphite sheet electrodes and a PMMA tube containing 0.4 M K_3_Fe(CN)_6_/K_4_Fe(CN)_6_ electrolyte. Copper (Cu) wires and thermal couples were inserted into each electrodes for measurement of electric signals and temperature, respectively. For the device labelled as PPy-PCM, a polypyrrole modified graphite sheet (Figure 2(b)) with 3 cm diameter was used as the top electrode of TGC, and a Cu foam/PEG1000 container (diameter of 3 cm, height of 6 cm) was connected to the bottom electrode, as shown in Figure S1a. In comparison, the devices labelled as G-PCM (using graphite and PCM) and G-blank (using only graphite) used the same-size pristine graphite sheets (Figure 2(b)) as both the top and the bottom electrodes. Furthermore, a large-scale prototype module was fabricated with a 10 cm x 10 cm polypyrrole-modified graphite sheet (Figure S10a) and a Cu foam/PEG1000 container (size of 10 cm x 10 cm, height of 6 cm) via the same processes. Finally, as shown in Figure S1b, all devices were fixed with an aluminium foil-covered foam box for the following performance tests to eliminate thermal losses and avoid the interference of extra sunlight absorption around the devices with the experimental results.

**Material characterization**

The morphology and structure of PPy/graphite and graphite were characterized by scanning electron microscopy (SEM, FEI Nova Nano 450). The FTIR spectra of PPy/graphite, pristine PPy and graphite were characterized by FTIR spectrometry (Bruker Vertex 70). The absorptivity/emissivity spectra of PPy/graphite and graphite in both the solar (0.3 to 2.5 μm) and infrared (2.5 to 25 μm) regions were characterized by UV-Vis-NIR spectrophotometry (Shimadzu UV-3600) and FTIR spectrometry (Bruker Tensor 27), respectively. We used integrating spheres to account for the scattered light from the full solid angle in both spectral regions [2]. The thermal gravimetric analysis (TGA) profile (from room temperature to 700 °C) of as-prepared PPy was characterized by thermogravimetric analysis (PerkinElmer Pyris1 TGA) with a temperature scan rate of 10 °C min^-1^. The heat flux per unit mass versus temperature from -5 °C to 80 °C was analysed by differential scanning calorimetry (PerkinElmer Diamond DSC) with a temperature scan rate of 5 °C min^-1^.

**Device characterization**

The solar light was supplied by a solar simulator (Newport). Different light intensities were obtained by a collecting lens. The IR images of the samples under solar illumination were captured by an infrared camera (Fluke TiX520). Thermocouples (OMEGA TT-K-36-SLE) and a data logger (Pico USB-TC-08) were used to measure temperature in all experiments. The ambient relative humidity was recorded by a humidity meter (Center 310 Rs-232). The open-circuit voltage (*V*_oc_) and the current-voltage (*I-V*) curves of all devices were measured by a Keithley 2000 multi-meter and Keithley 2400 instrument, respectively.

**Supplementary Note 1: Thermal storage material (Cu foam/PEG1000) simulations**

According to previous literature, the thermal effusivity (*e*), the measure of a material’s ability to transfer thermal energy with its surroundings, is defined as follows: [3]

$e=\sqrt{kC}=\sqrt{k\rho C_{p}}$ (1)

where *k* is the thermal conductivity, *C* is the volumetric heat capacity, *ρ* is the density and *C_p_* is the specific heat. However, in practical operation of phase change materials (PCMs), effective thermal effusivity (*e_eff_*) is often used as an alternative:

$e_{eff}=\sqrt{k\rho h}$ (2)

where *h* is the latent heat per unit mass. The upper bound of the thermal conductivity for an isotropic, two-phase composite can be estimated by the Hashin-Shtrikman (HS) model, which necessarily neglects the presence of interfacial resistance between the two phases of the composite [4]. Based on the HS model, the theoretical thermal conductivity of Cu foam/PEG1000 can be estimated as shown:

$k_{HS}=k_{Cu}\left[ \frac{2k_{Cu}+k_{PEG1000}-2\left( k_{Cu}-k_{PEG1000} \right)\varphi}{2k_{Cu}+k_{PEG1000}+\left( k_{Cu}-k_{PEG1000} \right)\varphi} \right]$ (3)

where *k_Cu_* is the thermal conductivity of pure copper (Cu, 385.7 W m^-1^ K^-1^) [1], *k_PEG1000_* is the thermal conductivity of pure PEG1000 (0.188 W m^-1^ K^-1^) [5], and *φ* is the porosity of Cu foam (95%). The volumetric latent heat of Cu foam/PEG1000 can be estimated as follows:

$\left( h\rho\right)_{eff}=\left( h\rho\right)_{PEG1000}\varphi$ (4)

where *h* and *ρ* of PEG1000 are 151 J g^-1^ (Figure S6a) and 1.2 g cm^-3^, respectively. Furthermore, as shown in Figure S6b, the *e_eff_* of Cu foam/PEG1000 can be estimated by combining Supplementary Equations 2, 3 and 4:

$e_{eff}=\sqrt{\left( h\rho\right)_{PEG1000}\varphi k_{Cu}\left[ \frac{2k_{Cu}+k_{PEG1000}-2\left( k_{Cu}-k_{PEG1000} \right)\varphi}{2k_{Cu}+k_{PEG1000}+\left( k_{Cu}-k_{PEG1000} \right)\varphi} \right]}$ (5)

For practical outdoor operation of the thermogalvanic generator, the optimal volume of Cu foam/PEG1000 must be estimated. Considering only longitudinal heat conduction, we simplifies the Cu foam/PEG1000 as a continuous linear, 1-D system, and the corresponding partial differential equation of heat conduction is defined as follows:

$\frac{\partial T(l,t)}{\partial t}=\alpha\frac{\partial^{2}T(l,t)}{\partial l^{2}}$ (6)

where *T*(*l,t*), *α* and *l* are the temperature, thermal diffusivity and spatial dimension of Cu foam/PEG1000, respectively. The thermal diffusivity (*α*) can be calculated by *α=k*/*ρc*. However, for a practical process with temperature fluctuation (*∆T*) around the phase transition temperature (*T**) of Cu foam/PEG1000, an apparent thermal diffusivity (*α_app_=k∆T*/*ρh*) was used as an alternative for *α* in the calculation [1]. Considering the initial temperature of Cu foam/PEG1000 (*T*_0_ = *T**-∆*T*) and the thermal flux (*q_0_*) at the interface between Cu foam/PEG1000 and the thermogalvanic cell, the boundary conditions can be given as follows:

$T\left( l,0 \right)=T_{0}$ (7)

$-k\left. \frac{\partial T\left( l,t \right)}{\partial l} \right|_{l=0}=q_{0}$ (8)

It is obvious that this system is similar to a body heated only by constant thermal flux. To solve the above equations, a semi-infinite body model is adopted for this system. Using separation of variables, the time and space distribution for the temperature profile of Cu foam/PEG1000 is solved as shown:

$T\left( l,t \right)=T_{0}+\frac{2q_{0}\sqrt{\frac{\alpha t}{\pi}}}{k}exp\left( -\frac{x^{2}}{4\alpha t} \right)-\frac{q_{0}l}{k}erfc\left( \frac{x}{2\sqrt{\alpha t}} \right)$ (9)

We assume that the optimal volume of Cu foam/PEG1000 depends only on absorption of all of the residual solar thermal energy during the daytime, i.e., meaning that all of the Cu foam/PEG1000 undergoes a phase change under solar illumination. If the length along the thermal flux of the Cu foam/PEG1000 is *L*, the optimal *L* can be described as follows:

$T\left( L,t \right)=T^{*}$ (10)

Considering the effective operation time under the solar illumination of a day to be approximately 8 h, the absolute term *|T_L_-T*|* is a function of thermal flux and the length of Cu foam/PEG1000. Furthermore, *|T_L_-T*|* can be used to estimate the optimal *L* for Cu foam/PEG1000 in the simulation, as shown in Figure S9b.

**Supplementary Note 2: Energy efficiency analysis**

For a TGC system, the maximum output power can be determined from the current-voltage (*I-V*) curves:

$P_{max}=\frac{1}{4}V_{oc}\times I_{sc}$ (11)

where *V_oc_* and *I_sc_* are the open-circuit voltage and short-circuit current, respectively. The energy conversion efficiency (*η_r_*) relative to the Carnot efficiency limit of a heat engine is defined as follows:

$\eta_{r}=\frac{\eta}{\left( \Delta T/T_{H} \right)}=\frac{P_{max}/\left( k\frac{\Delta T}{d} \right)}{\left( \Delta T/T_{H} \right)}$ (12)

where the thermal conductivity (*k*) of the 0.4 M K_3_Fe(CN)_6_/K_4_Fe(CN)_6_ electrolyte is 0.57 W m^-1^ K^-1^ [6], the electrode separation distance (*d*) is 3 cm, the hot-side temperatures (*T_H_*) are respectively 348 K and 310 K for the PPy/graphite device under one-sun illumination and after illumination, and the corresponding values of *∆T* are 38.7 K and 13 K, respectively. Hence, the values of *η_r_* for the PPy/graphite device under one-sun illumination and after illumination are calculated as 0.73% and 0.51%, respectively.

For all of the thermogalvanic generators discussed in this work, the total input energy into the devices is solar energy. The total efficiency (*η_total_*) of the thermogalvanic generators can be expressed as follows:

$\eta_{total}=\frac{P_{max}}{C_{opt}q_{i}}$ (13)

where C_opt_ is the optical concentration, and *q_i_* is the nominal direct solar irradiation of 1 kW m^-2^. The total efficiency (*η_total_*) values of all devices are shown in Figure S8.

**Supplementary figures**

**
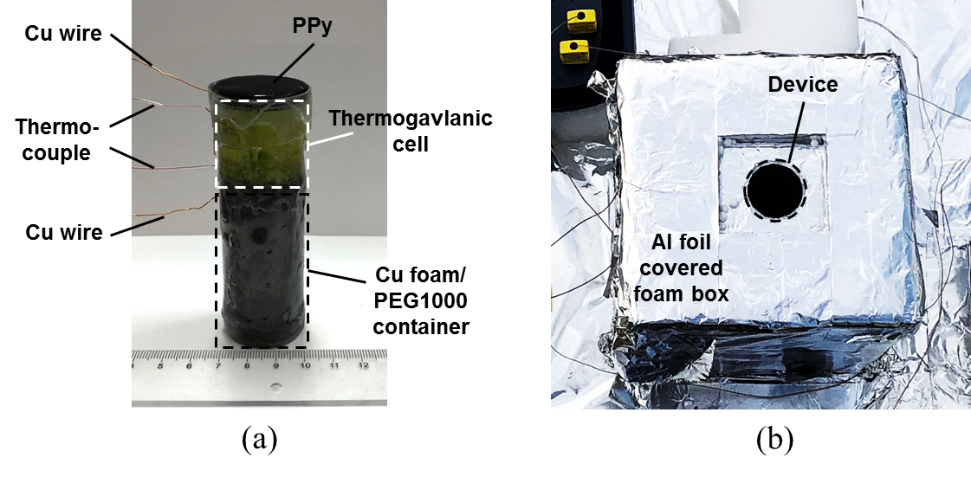
**

Figure S1: Photographs of the thermogalvanic cell. (a) The device consists of a PPy layer, thermogalvanic cell and Cu foam/PEG1000 container. The top electrode and bottom electrode are connected with both thermocouples and Cu wires to measure the temperature and electric signals, respectively. (b) In all experiments, both the device and comparisons are fixed with aluminium foil-covered foam boxes to decrease thermal losses and avoid extra sunlight absorption around the devices.

**
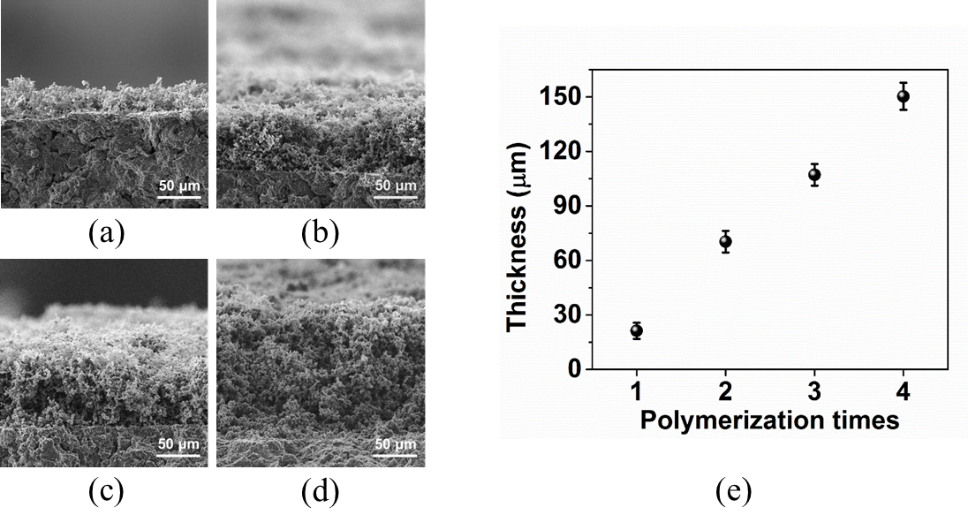
**

Figure S2: Characterization of the thickness of PPy. (a-d) Cross-sectional SEM images of PPy-modified graphite sheet with different polymerization times. (e) Average thickness of PPy layer versus polymerization times.

**
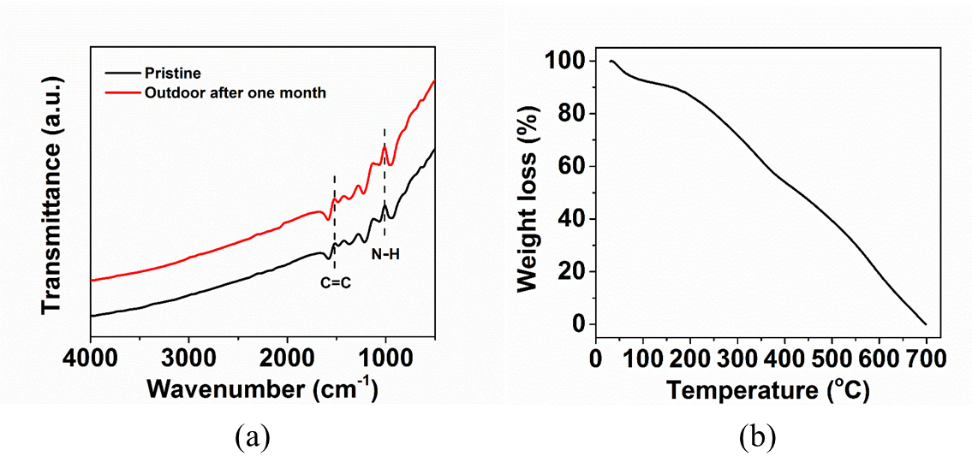
**

Figure S3: Chemical and thermal stability of PPy. (a) FTIR spectra of PPy/graphite exposed to the environment after one month and pristine samples. All characteristic peaks of PPy, without any shifts and new signals, present the excellent chemical stability of the prepared PPy/graphite. (b) TGA profile of as-prepared PPy. The initial weight loss from 100 to 230 °C is attributed to the evaporation of physically adsorbed water molecules and the removal of unreacted monomers from the surface. The second weight loss from 230 °C to 700 °C corresponds to the disintegration of PPy polymer chains. The excellent thermal stability satisfies the criteria for operation below 100 °C under natural sunlight.

**
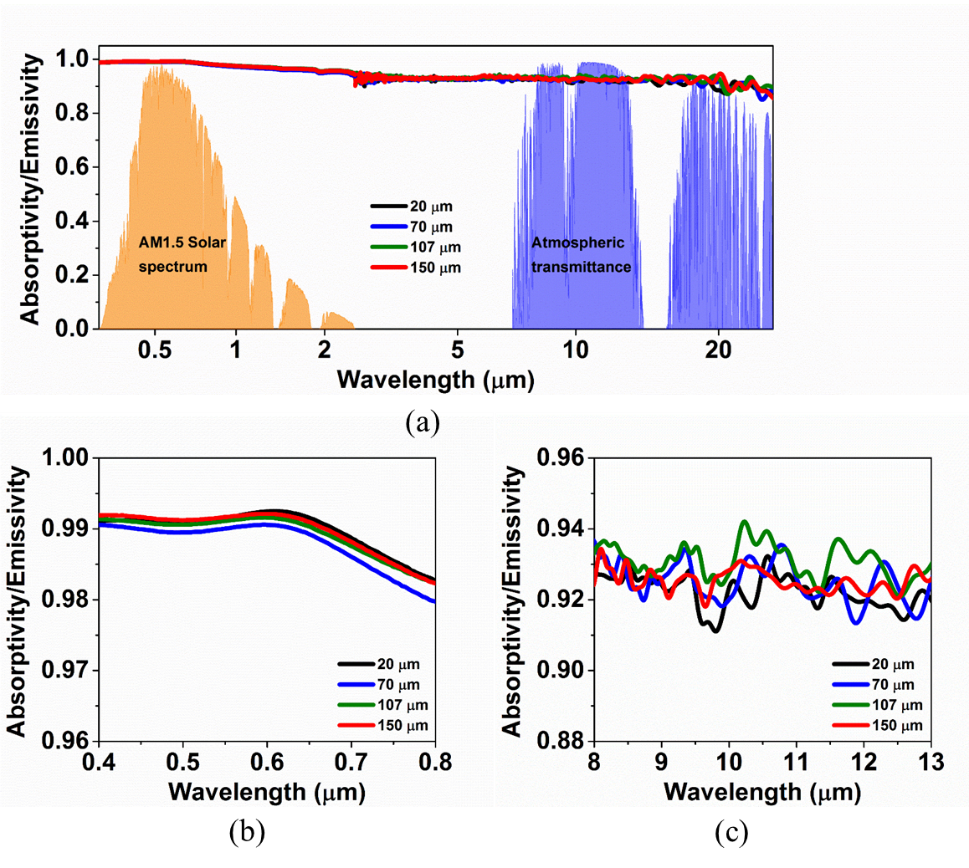
**

Figure S4: Absorptivity/emissivity spectrum of PPy/graphite with different PPy thicknesses. (a) Broadband absorptivity/emissivity spectrum from 0.3 to 25 μm containing both solar and infrared regions with the standard AM1.5 solar spectrum (shaded orange) and atmospheric transmittance *t*(*λ*) (shaded blue) plotted for reference [7]. (b) Magnified spectrum over the visible region (0.4-0.8 μm containing most of the solar irradiation) with a maximum absorptivity/emissivity of 0.993. (c) Magnified spectrum over the atmospheric LWIR transmission window (8-13 μm, which determines the performance of radiative cooling) with an average absorptivity/emissivity as high as 0.93. It is worth noting that all of the absorptivity/emissivity values with different PPy thicknesses are nearly equal within the range of errors.

**
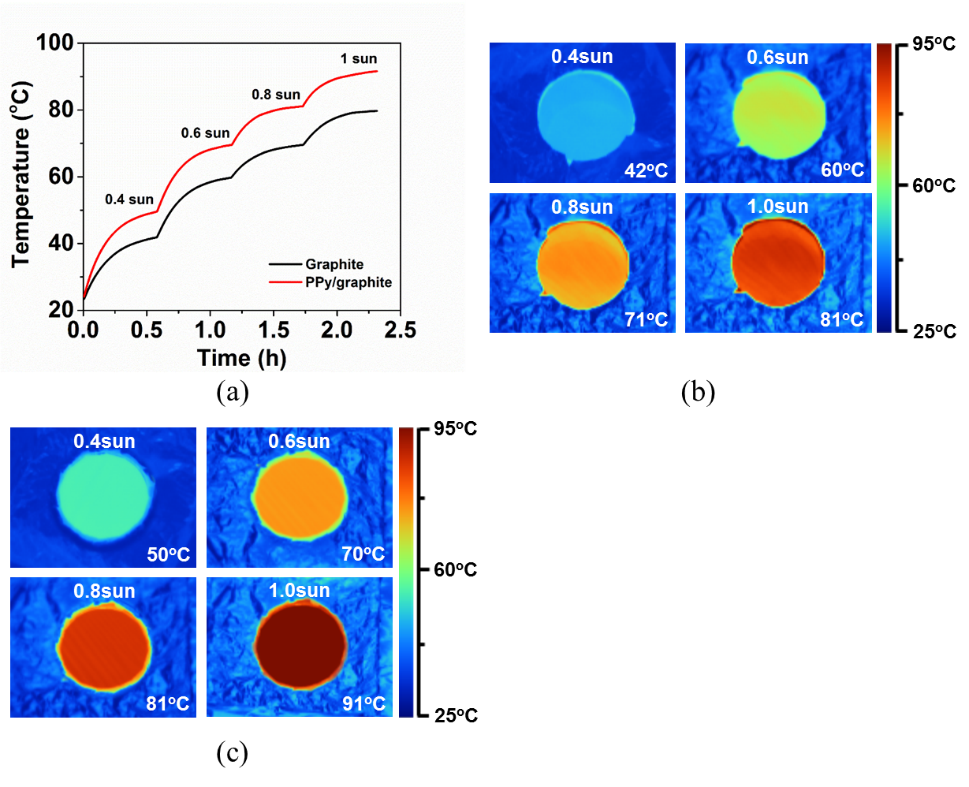
**

Figure S5: Performance of heating for PPy/graphite and graphite. (a) Increasing temperature curve of PPy/graphite and graphite according to increments in solar intensity from 0.4 to 1 kW m^‑2^ (labelled as 0.4 sun to 1 sun). IR thermal images of PPy/graphite (b) and graphite (c) at steady state with 0.4 to 1 sun.

**
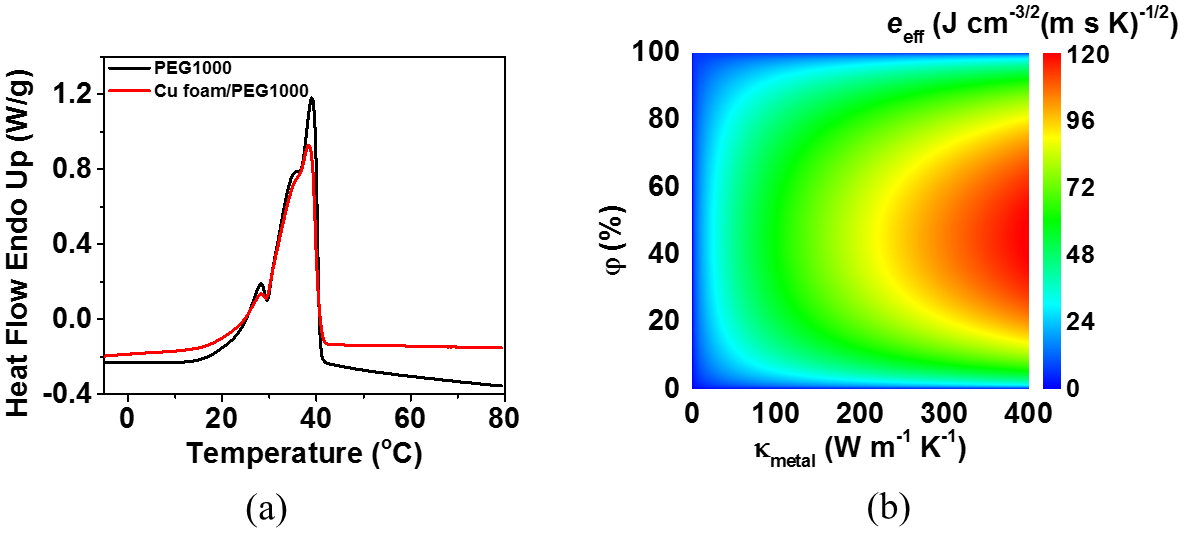
**

Figure S6: Characterization and simulation of Cu foam/PEG1000. (a) Heat flux per unit mass curves of Cu foam/PEG1000 and pure PEG1000 versus temperature. It can be observed that the phase transition temperature (*T**) of Cu foam/PEG1000 is near 38 °C, which is suitable for the device during all-day operation. The latent heat per unit mass (*h*) values of PEG1000 and Cu foam/PEG1000 are calculated as 151 J g^-1^ and 119 J g^-1^, respectively, by integrating the area. (b) Simulated maps of effective thermal effusivity (*e_eff_*) according to Supplementary Equation 5 plotted as a function of the metal foam porosity (*φ*) and pure metal thermal conductivity (*k_metal_*). For Cu foam/PEG1000 with *φ* of 95% and *k_Cu_* of 385.7 W m^-1^ K^-1^, the theoretical value of *e_eff_* is calculated as approximately 48 J cm^-3/2^(m s K)^-1/2^.

**
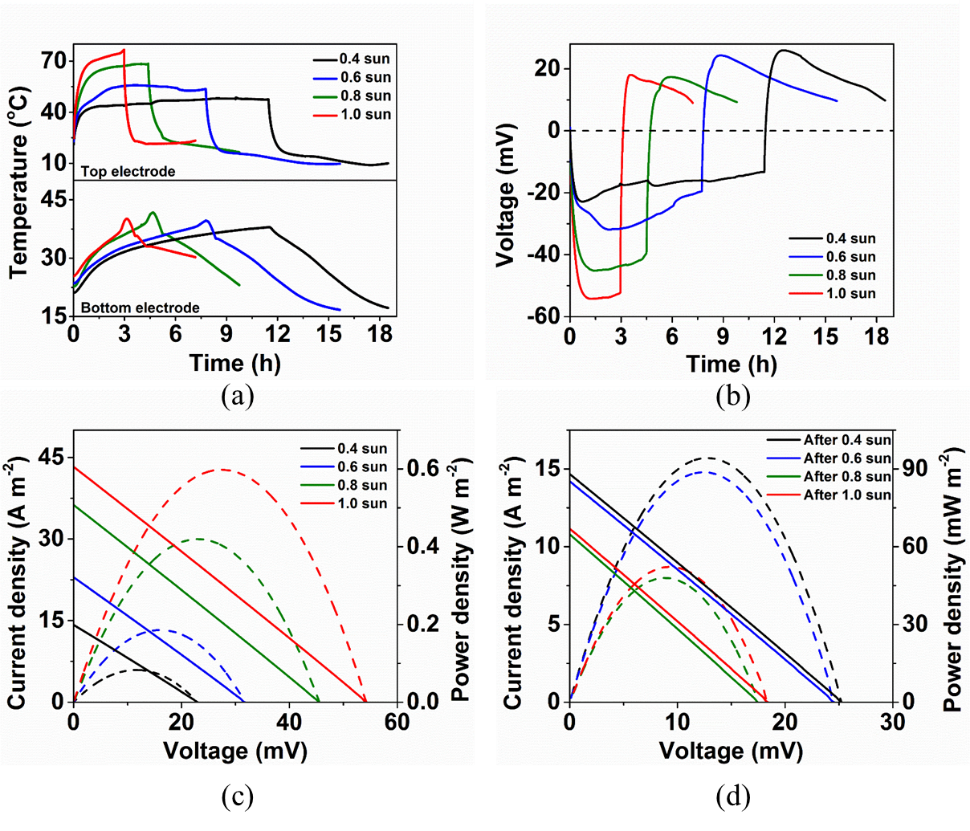
**

Figure S7: Performance of the device labelled as PPy-PCM. (a) Temperature curves of the top electrodes (upper) and the bottom electrodes (lower) and (b) corresponding open-circuit voltage (*V_oc_*) curves of the device versus time. Maximum current-voltage and power-voltage curves of the devices under illumination from 0.4 to 1 sun (c) and after illumination (d).

**
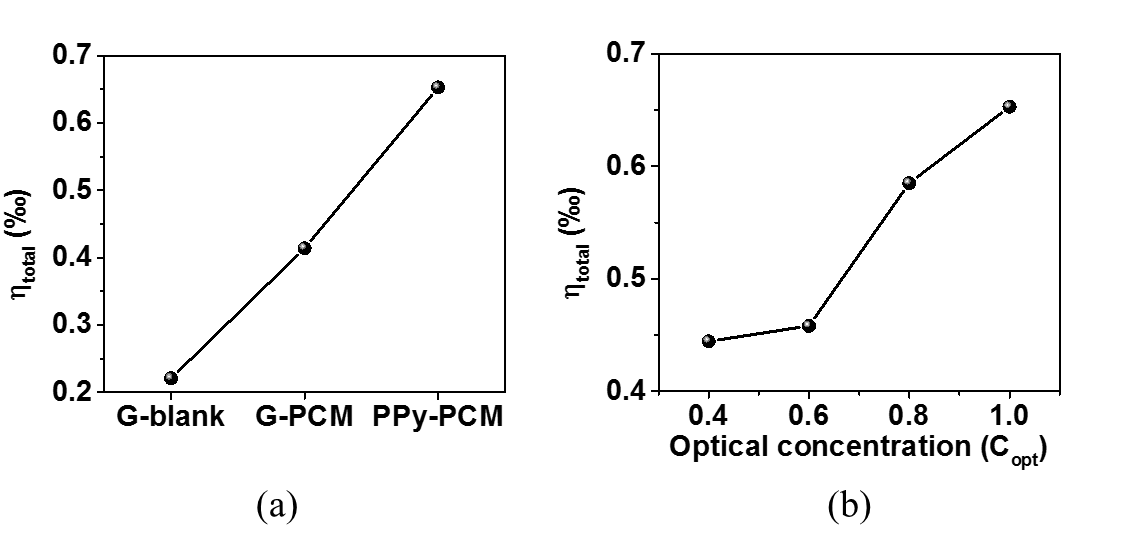
**

Figure S8: Efficiency analysis of the thermogalvanic generator. (a) Total efficiency (*η_total_*) of different thermogalvanic generators labelled as G-blank, G-PCM, and PPy-PCM under one-sun illumination and after illumination, respectively. (b) Total efficiency (*η_total_*) of PPy-PCM under illumination from 0.4 to 1 sun and after illumination. The total efficiency (*η_total_*) values for all devices are calculated using Supplementary Equation 13.

**
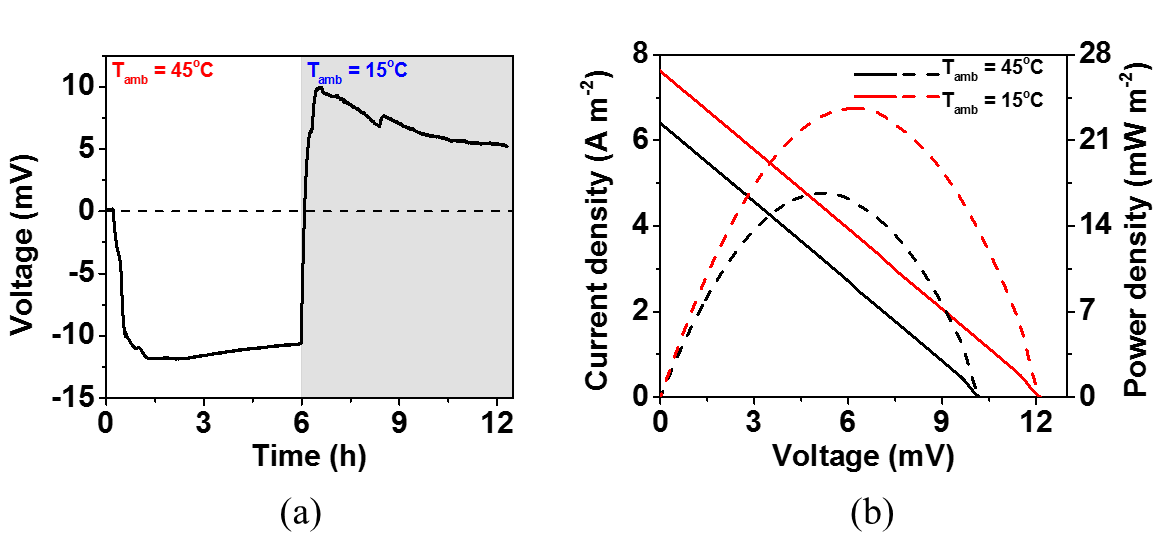
**

Figure S9: Performance of PPy-PCM exposed to hot and cold environment without illumination successively. (a) Open-circuit voltage (*V_oc_*) curves of the device at *T_amb_*=45 ^o^C (white area) and *T_amb_*=15 ^o^C (gray area). (b) Maximum current-voltage and power-voltage curves of the device at *T_amb_*=45 ^o^C and *T_amb_*=15 ^o^C, respectively.

**
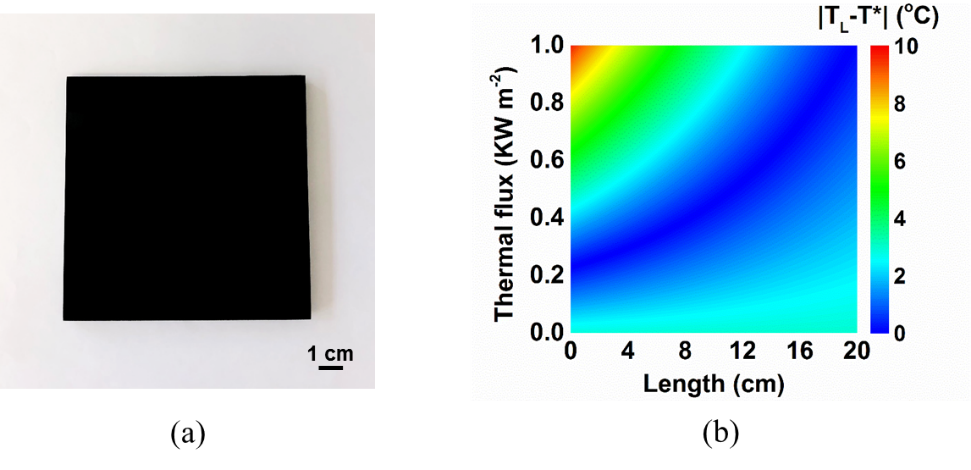
**

Figure S10: Preparation for a large-scale prototype module. (a) Photograph of large-scale PPy/graphite with an active area of 10 cm x 10 cm. (b) Simulated maps of |T_L_-T*| (the absolute value of the differential between temperature at the length and the phase transition temperature) according to Supplementary Equations 9 and 10 plotted as a function of the thermal flux and the length of Cu foam/PEG1000. To estimate the optimal volume of Cu foam/PEG1000, we assume that the effective operation time under solar illumination for a day is 8 h and treat |T_L_-T*|=0 as the complete phase transition of Cu foam/PEG1000.

**Supplementary References**

[1] A. L. Cottrill, A. T. Liu, Y. Kunai et al., "Ultra-high thermal effusivity materials for resonant ambient thermal energy harvesting," *Nature Communications*, vol. 9, no. 1, p. 664, 2018.

[2] Y. Zhai, Y. Ma, S. N. David et al., "Scalable-manufactured randomized glass-polymer hybrid metamaterial for daytime radiative cooling," *Science*, vol. 355, no. 6329, p. 1062, 2017.

[3] J. A. Balderas-Lopez, D. Acosta-Avalos, J. J. Alvarado et al., "Photoacoustic measurements of transparent liquid samples: thermal effusivity," *Measurement Science and Technology*, vol. 6, no. 8, p. 1163, 1995.

[4] P. Kumar, and F. Topin, "Thermal conductivity correlations of open-cell foams: Extension of Hashin–Shtrikman model and introduction of effective solid phase tortuosity," *International Journal of Heat and Mass Transfer*, vol. 92, pp. 539-549, 2016.

[5] F. Agyenim, N. Hewitt, P. Eames, and M. Smyth, "A review of materials, heat transfer and phase change problem formulation for latent heat thermal energy storage systems (LHTESS)," *Renewable Sustainable Energy Review*, vol. 14, no. 2, pp. 615-628, 2010.

[6] J. Duan, G. Feng, B. Yu et al., "Aqueous thermogalvanic cells with a high Seebeck coefficient for low-grade heat harvest," *Nature Communications*, vol. 9, no. 1, p. 5146, 2018.

[7] "IR Transmission Spectra," accessed October 12, 2018, <http://www.gemini.edu/?q=node/10789>
